# MiRNAs with Apoptosis Regulating Potential Are Differentially Expressed in Chronic Exercise-Induced Physiologically Hypertrophied Hearts

**DOI:** 10.1371/journal.pone.0121401

**Published:** 2015-03-20

**Authors:** Subbiah Ramasamy, Ganesan Velmurugan, K. Shanmugha Rajan, Tharmarajan Ramprasath, Krishnan Kalpana

**Affiliations:** 1 Cardiac Hypertrophy Laboratory, Department of Molecular Biology, School of Biological Sciences, Madurai Kamaraj University, Madurai, Tamilnadu, India; 2 Department of Medicine, University of Alberta, Edmonton, Alberta, Canada; 3 Department of Plant Pathology, Regional Research Station, Tamilnadu Agricultural University, Krishangiri, Tamilnadu, India; Texas A& M University Health Science Center, UNITED STATES

## Abstract

Physiological cardiac hypertrophy is an adaptive mechanism, induced during chronic exercise. As it is reversible and not associated with cardiomyocyte death, it is considered as a natural tactic to prevent cardiac dysfunction and failure. Though, different studies revealed the importance of microRNAs (miRNAs) in pathological hypertrophy, their role during physiological hypertrophy is largely unexplored. Hence, this study is aimed at revealing the global expression profile of miRNAs during physiological cardiac hypertrophy. Chronic swimming protocol continuously for eight weeks resulted in induction of physiological hypertrophy in rats and histopathology revealed the absence of tissue damage, apoptosis or fibrosis. Subsequently, the total RNA was isolated and small RNA sequencing was executed. Analysis of small RNA reads revealed the differential expression of a large set of miRNAs during physiological hypertrophy. The expression profile of the significantly differentially expressed miRNAs was validated by qPCR. *In silico* prediction of target genes by miRanda, miRdB and TargetScan and subsequent qPCR analysis unraveled that miRNAs including miR-99b, miR-100, miR-19b, miR-10, miR-208a, miR-133, miR-191a, miR-22, miR-30e and miR-181a are targeting the genes that primarily regulate cell proliferation and cell death. Gene ontology and pathway mapping showed that the differentially expressed miRNAs and their target genes were mapped to apoptosis and cell death pathways principally *via* PI3K/Akt/mTOR and MAPK signaling. In summary, our data indicates that regulation of these miRNAs with apoptosis regulating potential can be one of the major key factors in determining pathological or physiological hypertrophy by controlling fibrosis, apoptosis and cell death mechanisms.

## Introduction

Cardiac hypertrophy is an adaptive response of the heart, during which terminally differentiated cardiomyocytes increase in size without undergoing cell division [1]. Cardiac hypertrophy is classified as physiological and pathological hypertrophy. Physiological hypertrophy (athlete's heart) is induced in response to chronic exercise training and pregnancy. It is reversible and characterized by normal cardiac morphology and function [2, 3]. Heart mass in professional athletes assessed by echocardiography demonstrates significantly increased value with similar diastolic and systolic functions compared with sedentary age-matched control individuals [4]. In contrast, pathological hypertrophy induced during onset of diseases is associated with increased interstitial fibrosis, apoptosis, switch from oxidative to glycolytic metabolic profile and cardiac dysfunction [2]. Numerous experimental and epidemiological studies explained that exercise conditioning can reverse or delay the onset of myocardial infarction and cardiomyopathy [5] and hence, physiological hypertrophy is considered as a positive sign to prevent cardiac dysfunction and failure.

The extracellular signal and the molecular signaling pathways associated with pathological and physiological hypertrophy was discussed by McMullen and Jennings [6] and Bernado *et al*. [7]. The insulin-like growth factor-1 (IGF1)/ phosphaoinositide-3-kinase (PI3K) pathway and G_αq_ pathway play a distinct role in induction of physiological and pathological hypertrophy respectively [6]. The hallmark molecular feature of physiological cardiac hypertrophy is the absence of induction of fetal genes, apoptotic, fibrotic and calcium signaling and continuation of fatty acid oxidation for ATP production [3]. Similar to the protein coding genes, microRNAs (miRNAs) that are small, noncoding RNAs (18–23 nt) involve in post transcriptional silencing of mRNAs by translational repression or cleavage were proven as key regulators in cardiac hypertrophy. In the last decade, different groups uncovered the role of miRNAs in pathological hypertrophy and categorized them as pro- and antihypertrophic miRNAs [8]. In contrast, very few studies reported the role of selected miRNAs during physiological hypertrophy [9–12]. Hence, the current study is aimed at global miRNA expression profiling to understand the role of miRNAs expressed during physiological cardiac hypertrophy. We found that miR-99, miR-100, miR-208, miR-181, miR-19 and many others were associated to cardiac hypertrophy and apoptosis. Furthermore, direct association of these downregulated miRNAs along with increased expression of the target gene expression was also observed in physiological hypertrophy models. Our study explored the significant differential expression of miRNAs with apoptosis regulating potential through PI3K/Akt/mTOR and MAPK signaling during physiological cardiac hypertrophy.

## Materials and Methods

### Animals

Wistar albino rats were maintained in the animal house at 25°C with 12 h day/night cycle. The animals were fed with deionized water and standard rat chow (Hindustan Lever Limited, India) *ad libitum*. The level of care provided to the animals met the basic requirements outlined in the guidelines formulated by National Institute of Health, USA. The animal protocols performed in this study were approved by the Internal Research and Review Board, Ethical Clearance, Biosafety and Animal Welfare Committee of Madurai Kamaraj University.

### Chronic Swimming Protocol

Swimming training protocol for rats was adopted from Balakumar and Singh [13] with some modifications. Briefly, eight-week old rats were allowed to swim for 90 minutes twice daily for eight weeks continuously. On the first day, the rats were subjected to swimming for 5 minutes twice, 10 minutes twice on the second day and thereafter the swimming time was increased at the rate of 10 minutes per day and reached the 90 minutes twice daily on ninth day and continued. The control rats were swam for 5 minutes twice weekly to compensate the water stress.

### Assessment of Physiological Hypertrophy

After eight weeks of training, the animals were anesthetized using ketamine (100 mg/ kg body weight) and the blood was collected by cardiac puncture. Subsequently, the heart was dissected out and perfused with sterile phosphate buffered saline. The Heart weight/ body weight ratio was measured. The cardiac tissue was fixed with 10% formaldehyde and paraffin embedded for histology analysis by standard methods. Subsequently, the embedded tissues were sliced into 5 μm thin sections using microtome. The sections were stained with hemotoxylin and eosin and mounted on slides. Finally, the tissue sections were observed for morphological changes and presence of apoptosis, necrosis or edema by microscopy with expertise from professional clinical pathologist. The images acquired by microscopy were employed for measurement of cell size using cell profiler software (www.cellprofiler.org) [14]. Appropriate settings were made to eliminate binucleated cells, fuzzy cells and incomplete cells at the borders. Minimum 500 cells each in biological triplicates were measured in every condition.

### Small RNA Sequencing

The harvested heart tissues were immediately frozen in liquid nitrogen and the total RNA was isolated using miRNEasy kit (Qiagen Inc., USA) and small RNA library was constructed using TruSeq kit (Illumina Inc., USA) as per manufacturers’ instructions. Small RNA sequencing was done commercially (Macrogen Inc., South Korea) with 50 bp single end reads in Illumina HiSeq 2000 machine. Raw data were processed using the Solexa software. Low quality reads were filtered according to the base quality value.

### Annotation to miRNAs

The miRNAs were identified and predicted from the Small RNA sequencing data by using miRDeep* program (http://www.australianprostatecentre.org/research/software/mirdeep-star) [15]. The reads were adapter trimmed and those reads less than 18–23 nucleotides were excluded from the analysis. Reads mapped to tRNA, snoRNA and piRNA were also excluded. Subsequently, the reads were aligned to rat genome (rn4) and tested for miRNA precursor stem loop structure with the strategy similar to miRDeep2 [16]. The predicted miRNAs were then annotated with miRbase and identified. The expression level of each miRNA is determined by the number of reads covering the mature miRNA region. Differential expression of identified miRNAs was calculated with R version 2.13.0 using DESeq version 1.4.1(http://www-huber.embl.de/users/anders/DESeq)[17]. The miRNA with read count of >1000, fold change of >1.5 and adjusted p-value with false discovery rate (FDR) < 0.1 were considered significant. The differential expression was analyzed statistically using Poisson distribution and the heat map was constructed.

### Prediction of miRNA Targets

The target genes of the respective miRNAs were predicted using the algorithms, including miRanda, TargetScan and RNAhybrid *via* miRwalk suite (http://www.umm.uni-heidelberg.de/apps/zmf/mirwalk) [18]. We restricted our search to minimum miRNA seed length of 7 nucleotides and binding sites on the 3' UTR of target mRNA. Targets predicted by at least two of the three algorithms with p≤0.05 were identified as predicted targets. The experimentally validated targets were identified by literature search and miRwalk.

### Validation of miRNA and Predicted mRNA Expression

The expression profile of significantly differentially expressed miRNAs and their respective target genes were validated by qRT-PCR. miRNA cDNA construction was carried out as described previously [19]. List of primers used for qRT-PCR quantification study of miRNAs are listed in S1 Table. For mRNA expression analysis, 1 μg of total RNA was used for cDNA construction using 100 units of M-MuLV reverse transcriptase (New England Biolabs, USA) as per manufacturer’s instructions in 20 μl reaction. Subsequently, qRT-PCR reactions were performed in duplicate in 10 μl final volume including 5 μl of 2X SYBR Green Master mix (Invitrogen, USA), 250 nM of each primer and 1 μl of a 1:10 dilution of the cDNA. Cycling conditions were 95°C for 10 minutes followed by 40 cycles of 95°C for 20 s, 60°C for 30s and 68°C for 30 s. A melting curve analysis (60°C to 99°C) was performed after the thermal profile to ensure specificity in the amplification. Primers used for qRT-PCR analysis of mRNA genes are provided in S2 Table. U87 and GAPDH genes were used as endogenous controls for normalization of miRNA and mRNA gene expressions respectively. Relative quantification (RQ) were calculated by 2^(−ΔΔCt)^ method and expressed as relative to endogenous controls.

### Gene Ontology and Pathway Mapping

To inspect the function of the differentially expressed miRNAs completely, we included the experimentally validated targets and performed gene ontology (GO) analysis using GO enrichment analysis and visualization tool (GOrilla) (http://cbl-gorilla.cs.technion.ac.il)[20] with a p-value threshold of 0.001. Functional annotation analysis was conducted using DAVID tools (http://david.abcc.ncifcrf.gov) to query KEGG pathways enriched with predicted miRNA targets. The analyses were conducted using the “fuzzy clustering algorithm” in order to reduce the redundancy among functionally related pathways that share similar target genes. Terms with Benjamini-corrected enrichment p-values <0.01 and FDR <0.05 were considered. Association map summarizing the enriched pathways was generated that provides a graphical representation reflecting the relationships between the terms based on the similarity of their target genes.

## Results and Discussion

**Fig 1:**
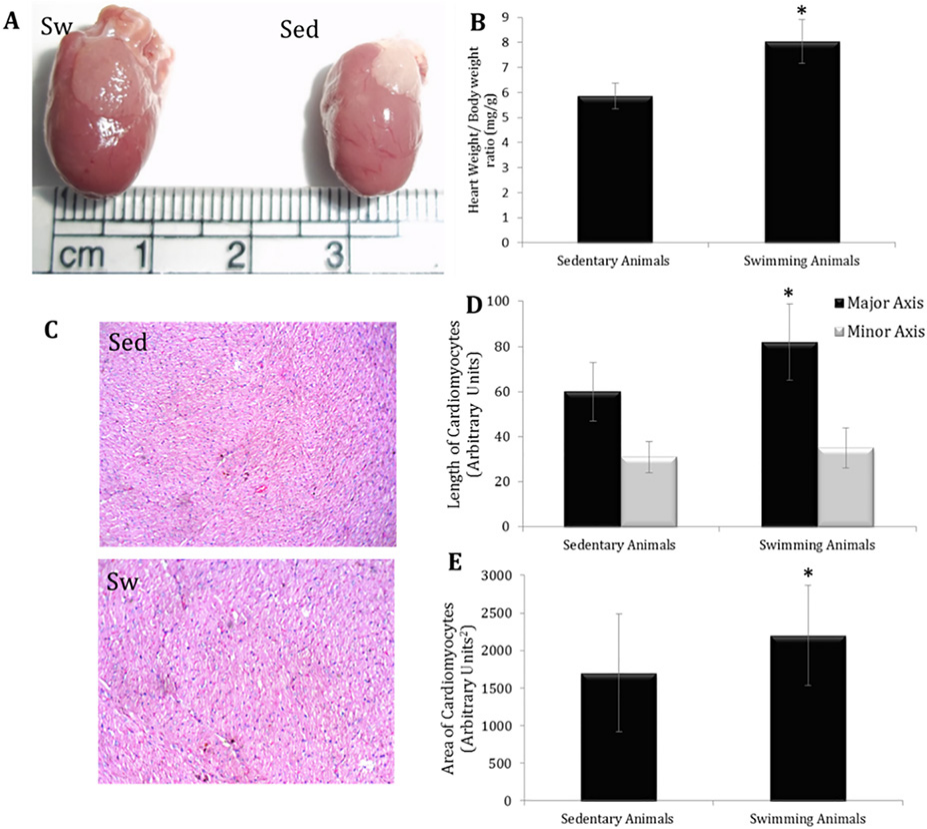
Assessment of induction of physiological cardiac hypertrophy. A. Representative photographs of the hypertrophied and control heart B. Heart weight/ body weight ratio. C. Representative histopathology images of hypertrophied and control cardiomyocytes. D. Cell profiler analysis of cell length and area of the cardiomyocytes. The values represent the mean±SD of six animals. Asterisk represents the statistical significance by Mann-Whitney U test at p-value < 0.005

Seven days continuous chronic swimming protocol for eight weeks successfully induced ~ 33% increase in the heart weight (Fig. 1A, B). Our protocol resulted in successful hypertrophy induction with increase in 1/3^rd^ of the heart weight that was achieved previously by adding weights to the swimming animals [21]. The histopathological results confirmed the hypertrophy induction without the signs of inflammation, necrosis or edema (Fig. 1C) which indicates that this hypertrophy can be considered as an adaptive protective mechanism to stress condition. Cell profiler analyses of the microscopic images revealed significant increase in cell length and area of cardiomyocytes (Fig. 1D, E).

**Fig 2:**
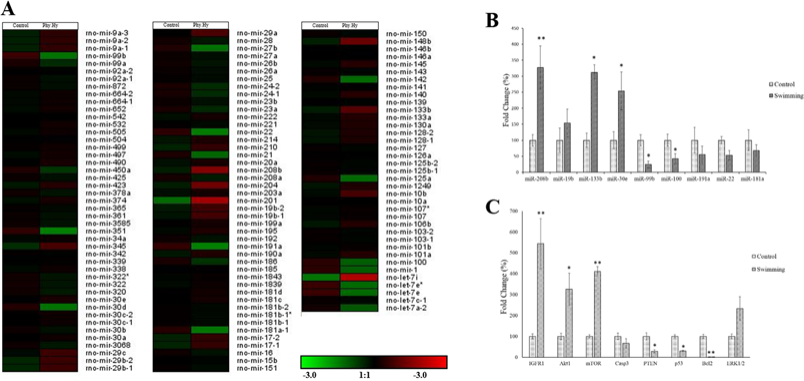
Expression profile of miRNAs and their target genes. A. Heat map representing the differential expression of miRNAs during physiological cardiac hypertrophy. miRNAs with higher read counts (> 1000) were considered for differential expression analysis. Analysis was statistically evaluated by Poisson distribution. B. Validation of expression profile of miRNAs by qPCR. U87 served as house keeping gene. Data represent the mean of biological triplicates with technical duplicates. Asterisk represents the statistical significance by One way ANOVA test at p-value <0.05. Double asterisk represents statistical significance at p-value < 0.005. C. Expression of target genes of miRNAs. The values were represented as mean±SE of three biological triplicates with technical duplicates. GAPDH served as house keeping gene. Asterisk represents the statistical significance by One way ANOVA test at p-value <0.05. Double asterisk represents statistical significance at p-value < 0.005.

Small RNA sequencing resulted in millions of small RNA reads that were mapped to rat genome and different RNA libraries. The homopolymer content, base call frequencies, base call qualities, Kmer enrichment andper sequence quality of the reads were found to be of good quality in all grounds. More than 80% of the miRNAs were differentially expressed (Fig. 2A). Out of 439 rat miRNAs reported in miRbase 19.0, 201 miRNAs showcased significant expression in our study. However, only the miRNAs with higher read count were taken into consideration. It is interesting to note that 63% of the miRNAs were downregulated during physiological hypertrophy. The differential expression of 128 miRNAs with read count more than 1000 was depicted in the form of heat map (Fig. 2A). 95 miRNAs were differentially expressed with fold change > 1.5 and adjusted p-value with FDR <0.1 (Fig. 2) and out of which 26 miRNAs had read count more than 10000. To be specifically mentioned (fold change >2.5), miR-208, miR-19b, miR-133b and miR-30e were significantly upregulated and miR-99b, miR-100, miR-191a, miR-22 andmiR-181a-1 were significantly downregulated. We validated the expression levels of selected significantly differentially expressed miRNAs by qRT-PCR. All the miRNAs displayed the similar pattern of up/ down regulation with variation in fold change on comparison to small RNA sequencing data. miR-208a, miR-133b and miR-30e showed more than 2.5-fold upregulation during physiological cardiac hypertrophy, while miR-19b showcased an upregulation of 1.5 fold only. miR-99b, miR-100, miR-191a, miR-22 and miR-181a were significantly downregulated during hypertrophy (Fig. 2B).

Targets of differentially expressed miRNAs during physiological hypertrophy were predicted by at least two of the three leading and reliable algorithms (TargetScan, RNAdb and miRanda) with p-value <0.05 were identified as predicted targets (S3 Table). The combination of these tools provides robust approach to identify high predictive, potentially functionally relevant miRNA-target gene interactions. A total of 1,682 genes were predicted by at least two of the three algorithms among which 272 target genes were common to all three (S1 Fig.). Subsequently, by literature search and miRwalk we enlisted the experimentally validated targets of miRNAs (S4 Table). The targets predicted by two of the three algorithms with p-value< 0.05 and experimentally validated targets were merged and used as a single dataset for subsequent analysis. To test the biological relevance of these predictions, we determined the levels of some of the important predicted target gene transcripts by qRT-PCR. In this analysis, the expression levels of the mRNAs were correlated with the expression level of corresponding miRNAs (Fig. 2C).

The summary of the miRNAs significantly differentially expressed are provided in Table 1. Our study revealed the significant downregulation of miR-99 family (Fig. 2A,B) that comprises of three miRNAs such as miR-99a, miR-99b and miR-100, which are present in 11, 1, 8 chromosomes respectively. The members of this family are the most downregulated miRNAs in our physiological hypertrophy model (Fig. 2A,B) and its target genes are upregulated (Fig. 2C). miR-100 was reported to be upregulated during heart failure by β-adrenergic receptor mediated repression of adult cardiac genes [22]. Recent evidence by Coppala *et al*.,[23] indicated that the miR-99a/let-7c cluster controls the cardiomyogeneic process by altering the epigenetic factors. This miR-99 family mainly targets *Akt/mTOR/IGF1* axis that leads to increased apoptosis and decreased protein synthesis during cancer. This is the first report to showcase the significant role of miR-99 family during cardiac hypertrophy. Another significantly downregulated candidate is miR-191a that belongs to the miR-191/425 cluster and abnormally expressed during different cancers, diabetes and Alzheimer disease. It regulates important cellular processes such as cell proliferation, differentiation, apoptosis and migration by targeting important transcription factors, chromatin remodelers and cell cycle associated genes [24]. There is no previous report on the expression of miR-191 in cardiac system. We found that the expression of miR-22 was also significantly downregulated during physiological cardiac hypertrophy (Fig. 2A,B). miR-22 is shown as sufficient to induce cardiomyocyte hypertrophy and key regulator of stress-induced cardiac hypertrophy and remodeling [25]. Our present study along with previously published reports, indicate that miR-22 plays a critical role in the regulation of cellular proliferation, differentiation and stress-induced hypertrophy (Table 1). miR-181a was reported to play a vital role during inflammatory response in cardiovascular system. Here in our study, the expression of this miRNA was downregulated during physiological cardiac hypertrophy and was predicted to target *MAPK1* and *TNF-α* genes. miR-181a protects the system from injury caused by inflammation by interfering the NF-κB functioning [26] and cell proliferation. Since there is no inflammatory response during physiological hypertrophy, expression of miR-181a was identified as downregulated (Fig. 2).

**Table 1:** List of significantly upregulated and downregulated genes and their target genes with previous studies in cardiac and other systems.

miRNA	Target Genes	This Study	Previous Reports in cardiac system
miR-19b	PTEN, MuRF,Bcl2, Atrogin-1, αCryB	miR-19b was significantly upregulated during physiological hypertrophy. The expression of predicted target genes such as PTEN, Bcl2 were validated by qRT-PCR.	Positively regulates cardiac hypertrophy by targeting Atrogin-1 and MuRF-1 [27]. It increases cell survival and negatively regulates apoptosis by targeting Bcl2
miR-30	CaMKIIδ, Egfr1, Bcl2	miR-30 was significantly upregulated during physiological hypertrophy. The expression of predicted target genes such as Bcl2 were validated by qRT-PCR.	angiotensin II induces down-regulation of miR-30 in cardiomyocytes, which in turn promotes myocardial hypertrophy through excessive autophagy [28]. miR-30 family was downregulated during pathological hypertrophy to activate calcium signaling, apoptosis and autophagy pathways
miR-133b	CyclinD, Nelf-A, RhoA, Ccd42	The expression of miR-133 was upregulated during physiological cardiac hypertrophy.	The expression of miR-133 was upregulated during pathological hypertrophy. A single infusion in vivo of an antagomir oligonucleotide suppressing miR-133 induced a marked and sustained cardiac hypertrophy [29].
miR-208b	THRAP1, Myostatin	The expression of miR-208b was upregulated during physiological cardiac hypertrophy.	Montgomery *et al*. [30] showed that therapeutic inhibition of miR-208a improves cardiac function and survival during heart failure.
miR-99b	IGF1R, Akt, mTOR	The expression of miR-99b was highly downregulated during physiological hypertrophy. The expression of its genes like IGF1R, Akt and mTOR were found to be upregulated during physiological hypertrophy.	Coppala *et al*.,[23] revealed that the miR-99a/let-7c cluster controls cardiomyogenesis process by altering the epigenetic factors.
miR-100	IGF1R, Akt, mTOR	The expression of miR-100 was highly downregulated during physiological hypertrophy. The expression of its genes like IGF1R, Akt and mTOR were found to be upregulated during physiological hypertrophy.	miR-100 was reported to be upregulated during heart failure by β-adrenergic receptor mediated repression of adult cardiac genes [22].
miR-191a	Egr1, Cd4, Casp4, SOCS4, p53	The exrepssion of miR-191a was downregulated during physiological hypertrophy. The expression of one of its target mRNA—p53 upregulation was confirmed by qRT-PCR.	No previous report on the role of miR-191a in cardiac system. miR-191a that belongs to the miR-191/425 cluster and abnormally expressed during different cancers, diabetes and Alzheimer disease.
miR-22	CDK6, Sir1, Sp1	The expression of miR-22 was downregulated during physiological hypertrophy	miR-22 is showed sufficient to induce cardiomyocyte hypertrophy and that miR-22 is a key regulator of stress-induced cardiac hypertrophy and remodeling [25]. miR-22 plays a critical role in the regulation of cellular proliferation, differentiation, and stress-induced hypertrophy
miR-181a	MAPK1, TNFα, GATA4	The expression of miR-181a was downregulated during physiological hypertrophy. miR-181a expression was induced during inflammation. Since, there is no inflammatory response during physiological hypertrophy—there is downregulation.	miR-181a was reported to play a vital role during inflammatory response in cardiovascular system. miR-181a protects the system from inflammation injury by interfering the NF- κB functioning [26] and cell proliferation.

We found the upregulation of miR-19b during physiological cardiac hypertrophy. This miR-19a/b family acts as a key regulator of cardiac hypertrophy and apoptosis. miR-19b positively regulates cardiomyocyte hypertrophy by targeting *atrogin-1* and *MuRF-1* and negatively regulates apoptosis through CN/NFAT–αCryB and Bim signaling under endoplasmic reticulum stress conditions [27]. Relative quantification of miR-19b expression was found to be upregulated along with downregulated expression of *Bcl2* (Fig. 2). Previous studies indicate that angiotensin II induces down-regulation of miR-30 in cardiomyocytes, which in turn promotes myocardial hypertrophy through excessive autophagy [28]. Our data extend these concepts by demonstrating an upregulation of miR-30 family in physiologically hypertrophied rat heart (Fig. 2A, B). Hence, it is logical to propose that the downregulated expression of miR-30 family in pathological hypertrophy activates calcium signaling, apoptosis and autophagy pathways.

**Fig 3:**
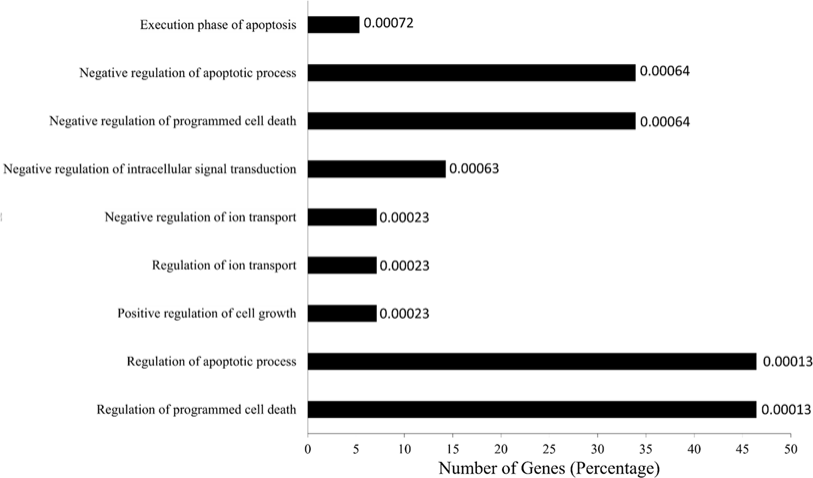
miRNA target genes were analyzed for GO enrichment and mapped for GO category. Percentage of target genes involved in each GO term. The represented GO terms were significant at p-value > 0.0001. The values at the end of the bars represent the p-values.

**Fig 4:**
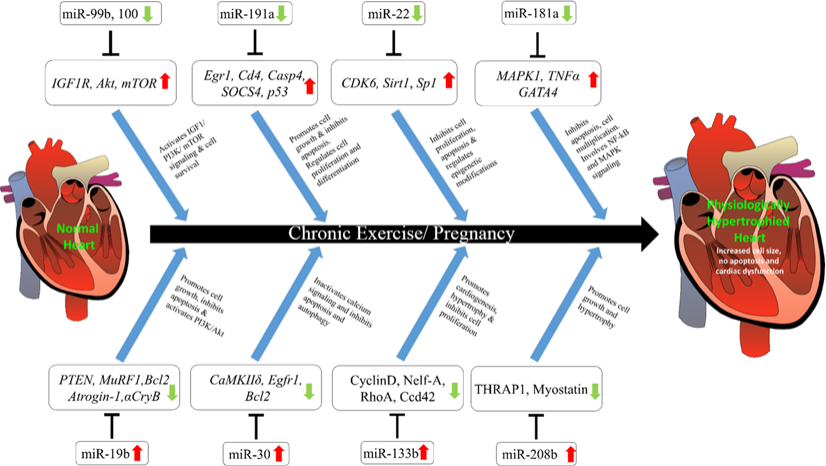
Regulation of physiological cardiac hypertrophy by miRNAs. Schematic representation of the miRNAs and their target genes in development of cardiac hypertrophy. The red and green arrow indicates the upregulation and downregulation respectively. The functions of the target mRNAs are also mentioned.

Our studies indicated the significant upregulation of the apoptotic miR-133b (Figs. 2, 3), which was reported to be downregulated during pathological hypertrophy [29]. A single infusion in vivo of an antagomir oligonucleotide suppressing miR-133 induced a marked and sustained cardiac hypertrophy [29]. Though miR-133 and miR-1 are bicistronic and reported to be jointly regulated during pathological hypertrophy, the expression of miR-1 was downregulated while miR-133 was upregulated in our case (Figs. 2, 4). This indicates the presence of regulatory mechanism of miRNA during post transcriptional downprocessing. In this study, during physiological cardiac hypertrophy we observed significant upregulation of miR-208a by both small RNA sequencing and qPCR (Figs. 2, 4). Montgomery *et al*. [30] showed that therapeutic inhibition of miR-208a improves cardiac function and survival during heart failure. The overall view of the miRNAs significantly regulated in our study and their target genes are provided in Fig. 4.

On submission of the target genes of the significantly differentially expressed miRNAs to GOrilla server with a p-value threshold of 0.001, new GO categories representing different processes were identified (S2 Fig.). Nearly 80% of the selected target genes were identified as involved in single cell-organism process, however being a multicellular organism study this ontology was not considered. It is interesting to note that approximately 50% of the target genes were in the regulation of apoptosis and cell death. While nearly 50% of the genes were involved in the regulation of protein metabolic process. Only very few genes (< 10%) were involved in negative regulation of intracellular transport and metal ion transport (Fig. 3).To better understand the predicted interactions, the experimentally and predicted validated targets were subjected to functional analyses using DAVID tools to query KEGG pathways enriched with the miR targets (S3 Fig.). We unraveled the major clusters containing more than a single pathway especially insulin-like growth factor (IGF1) signaling, PI3/Akt/mTOR, MAPK signaling and p53 signaling (S3 Fig.). Studies by Kemi *et al*. [31] proved that activation or inactivation of cardiac Akt/mTOR signaling diverges physiological hypertrophy from pathological hypertrophy. Our studies, a step ahead lead us to propose that regulation of these miRNAs to regulate apoptosis and cell death pathways may determine pathological or physiological hypertrophy (Fig. 4).

### Conclusion

Overall, our study indicates the critical role of miRNAs with potential to regulate apoptosis during cardiac hypertrophy and especially in regulating cell proliferation and cell death that determines the fate of cardiac hypertrophy *i*.*e*., pathological or physiological hypertrophy. Functional analysis of these miRNAs during both pathological and physiological hypertrophy and understanding of the regulatory mechanism of these miRNAs are necessary to explore its clinical utilizations. Exploration of these miRNAs will lead to therapeutic approaches to attenuate the maladaptive nature of pathological hypertrophy to positive nature of physiological hypertrophy and thereby this strategy can be an effective tool to prevent heart failure.

## Supporting Information

S1 Fig Venn diagram representing the number of targets of miRNAs predicted by three different programs. (TIF)

S2 Fig The geno ontology map of the significantly differentially expressed miRNAs. (TIF)

S3 Fig KEGG analysis pathway of the significantly differentially expressed miRNAs. (TIF)

S1 Table List of primer sequences used for the qPCR study of miRNAs. (XLSX)

S2 Table List of primer sequences used for the qPCR study of mRNAs. (XLSX)

S3 Table List of target genes predicted for the significantly, differentially expressed miRNAs by different programs. (XLSX)

S4 Table List of experimentally validated targets of the significantly, differentially expressed miRNAs. (XLSX)

S5 Table Mean Ct values of the miRNAs used for the normalization of qPCR analysis. (XLSX)

S6 Table Mean Ct values of the mRNAs used for the normalization of qPCR analysis. (XLSX)
