# Supplementary figures and images for: MiRNAs with Apoptosis Regulating Potential Are Differentially Expressed in Chronic Exercise-Induced Physiologically Hypertrophied Hearts
Source: PLoS One. 2015 Mar 20;10(3):e0121401. doi: 10.1371/journal.pone.0121401 (PMC4368613; doi:10.1371/journal.pone.0121401)

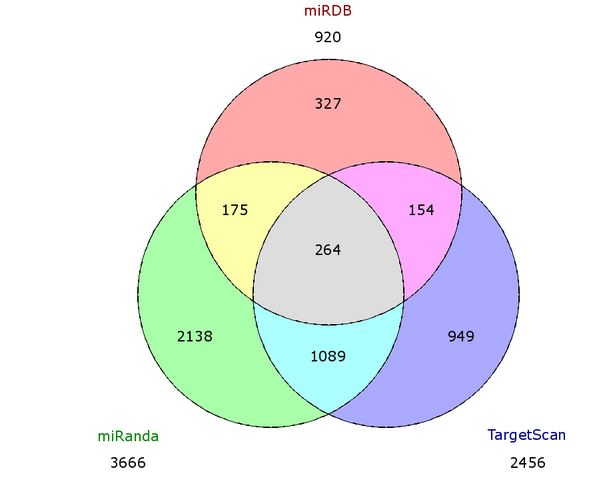

Supplement: S1 Fig — (TIF) [file pone.0121401.s001.tif]

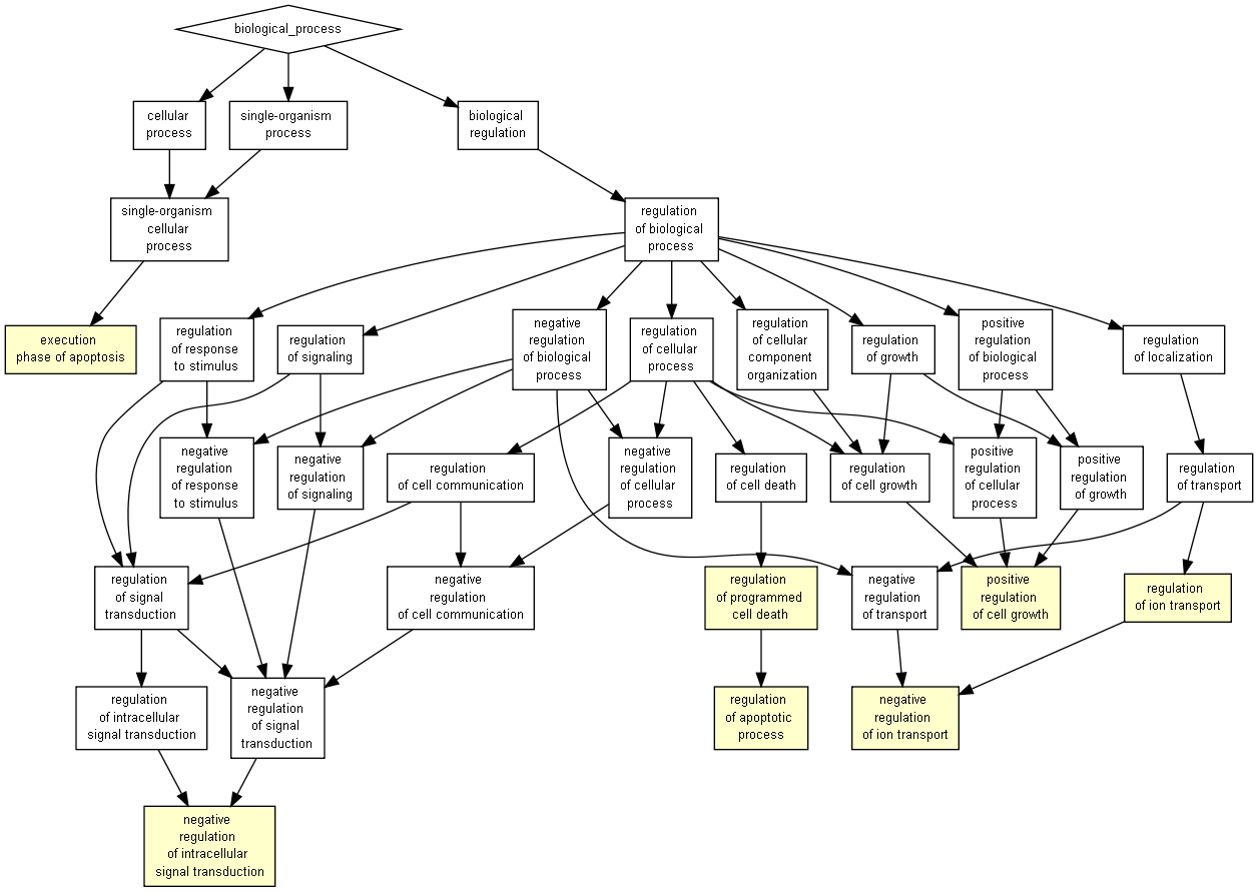

Supplement: S2 Fig — (TIF) [file pone.0121401.s002.tif]

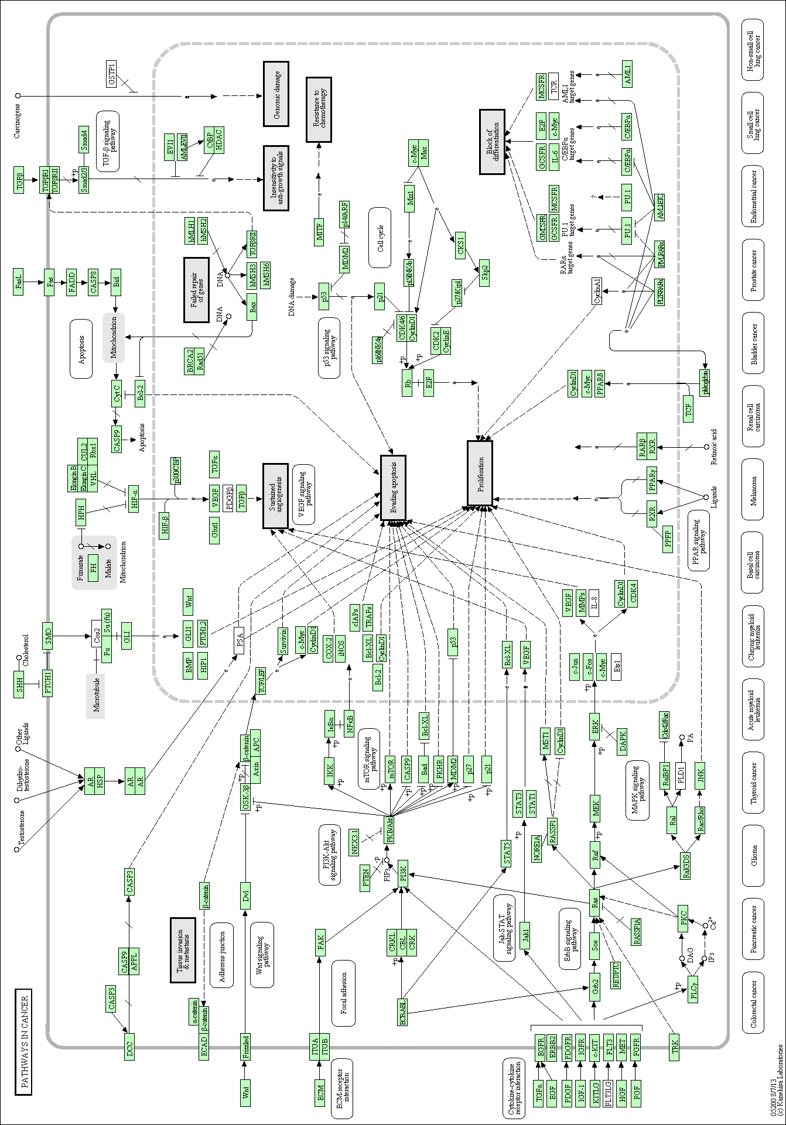

Supplement: S3 Fig — (TIF) [file pone.0121401.s003.tif]
